# Spontaneous Coronary Artery Dissection in a Patient With Cushing's Disease

**DOI:** 10.7759/cureus.36370

**Published:** 2023-03-19

**Authors:** Joana T Vieira, Bruno Besteiro, Ana Faceira, Pedro Marques, Jorge S Almeida

**Affiliations:** 1 Internal Medicine, Centro Hospitalar Universitário de São João, Porto, PRT

**Keywords:** spontaneous coronary artery dissection, cushing's syndrome, acute coronary syndrome, atypical spontaneous coronary artery dissection, hypercortisolism, coronary disease, cushing’s disease

## Abstract

Spontaneous coronary artery dissection (SCAD) is an uncommon cause of acute myocardial infarction, caused by a non-traumatic and non-iatrogenic separation of the coronary arterial wall, especially amongst young women with no conventional cardiovascular risk factors. We describe the case of a 46-year-old woman with a past medical history of Cushing's disease, treated surgically, who presented with SCAD. Cushing's disease is not considered a traditional risk factor for SCAD. As there are reported cases of arterial dissections associated with this entity and common causes of SCAD were excluded, we hypothesized that the patient's past medical history may have contributed to SCAD. To the best of our knowledge, there are no other reported cases of Cushing's disease-associated SCAD.

## Introduction

Spontaneous coronary artery dissection (SCAD) is a potential cause of myocardial infarction, with a prevalence in the general population of 1%-5% and is particularly common among the young [1-3]. It may affect both sexes, but approximately 90% of patients are women aged 47 to 53 years old. The burden of cardiovascular risk factors is lower in these cases when compared to patients with atherosclerotic coronary artery disease [4]. SCAD is a poorly understood phenomenon, but it seems to be associated with fibromuscular dysplasia (FMD), postpartum, multiparity, connective tissue disorders, systemic inflammatory conditions, and hormonal therapy [5,6]. Clinical manifestation is often acute coronary syndrome, but it can also present with ventricular arrhythmias or even cardiogenic shock [7].

In patients with Cushing's syndrome, cortisol has both direct and indirect effects on the vascular system [8, 9], and the higher mortality rate associated with this entity is mainly driven by cardiovascular complications [10]. In the literature, reported cases of aortic and vertebral arterial dissections, but not SCAD, were associated with Cushing's syndrome [9,11,12]. We describe the case of a 46-year-old woman with a history of Cushing's disease who presented with SCAD.

This article was previously presented as a meeting abstract at the European Society of Hypertension (ESH) and the International Society of Hypertension (ISH), held between April 11-14, 2021.

## Case presentation

A 46-year-old woman presented to the emergency department (ED) with chest pain that she felt while the patient was cooking. The pain started suddenly and radiated to the left arm. It lasted for 30 minutes, and the patient reported associated nausea and sweating. Previous episodes were mentioned but not deemed relevant by her.

**Figure 1:**
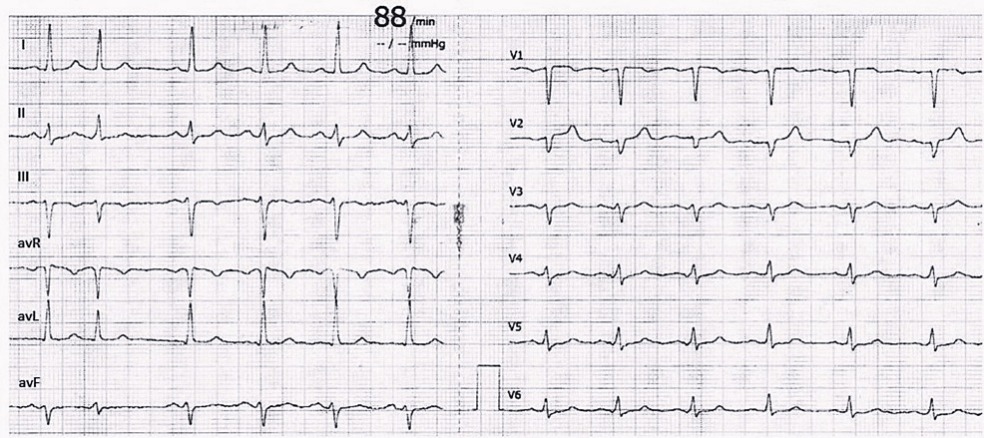
Electrocardiogram performed at admission

On examination, the blood pressure was 197/111 mmHg, the pulse was 89 beats per minute, and the oxygen saturation was 100% while the patient was breathing ambient air. An electrocardiogram showed sinus rhythm with poor R-wave progression in the precordial leads (Figure 1).

Table 1 shows the patient's laboratory test results.

**Table 1:** Laboratory test results Creatine kinase-MB: creatine kinase-myoglobin binding; TSH: thyroid stimulating hormone; T4: thyroxine; anti-Ds-DNA antibodies: anti-double stranded DNA antibodies

Variable	Day of admission	In hospital	Reference range
Hemoglobin (g/dl)	12.4	12.1	12.0-16.0
Hematocrit (%)	37.6	36.9%	37-49
Platelet count (per μl)	209,000	212, 000	150,000-400,000
White-cell count (per μl)	9.510	7.99	4.000-11.000
Neutrophils	54.4	58.0	53.8-69.8
Lymphocytes	36.2	32.0	22.6-36.6
Eosinophils	2.2	2.5	0.6-4.6
Sodium (mEq/liter)	140	139	135-147
Potassium (mEq/liter)	3.6	4.1	3.5-5.1
Chloride (mEq/liter)	105	106	101-109
Urea nitrogen (mg/dl)	30	47	10-50
Creatinine (mg/dl)	0.64	0.59	0-51-0.95
Glucose (mg/dl)	94	95	75-110
Calcium (mEq/L)	4.5	4.5	4.2-5.1
Magnesium (mEq/L)	1.94	1.85	1.55-2.05
Phosphorus (mg/dl)			2.7-4.5
C-reactive protein (mg/L)	1.2	3.6	<3.0
Hs-Troponin I (ng/L)	96.3 (maximum value)		<16
Myoglobin (ng/mL)	13.1	16.8	<146.9
Creatine kinase-MB (ng/ml)		0.4	0-6.4
B-type natriuretic peptide (pg/ml)		10.2	<100
Total protein (g/dl)		65.5	64.0-83.0
Albumin (g/dl)		38.3	38.0-51.0
Creatine kinase (U/liter)	108	50	10-149
Aspartate aminotransferase (U/liter)		14	10-31
Alanine aminotransferase (U/liter)		17	10-31
Alkaline phosphatase (U/liter)		58	10-31
Gamma-glutamyltransferase		32	7-32
Total bilirubin (mg/dl)		1.13	<1.2
TSH (UI/ml)		1.39	0.35-4.94
Free T4 (ng/dl)		1.05	0.7-1.48
Imunoglobulin G (mg/dl)		1120	650-1500
Imunoglobulin A (mg/dl)		109	78-312
Imunoglobulin M (mg/dl)		69	55-300
Complement C3c (mg/dl)		98	83-117
Complement C4 (mg/dl)		24	12-36
Antinuclear antibodies		Negative	< 1/100
Anti-Ds-DNA antibodies (UI/ml)		<10	<100
Antineutrophil cytoplasmic antibodies (U/ml)		<2	<20
Anticardiolipin antibodies		Negative	-
Citrullinated peptide antibodies (U/ml)		1.1	<7
Extractable Nuclear Antigen Antibodies		Negative	-
Rheumatoid factor (UI/ml)		11.6	<30
Lupus anticoagulant		Negative	
Hepatitis C virus		Non-reactive	
Hepatitis B virus		Previous infection	
Human immunodeficiency virus		Non-reactive	
Treponemal test		Negative	

Blood tests were unremarkable except for an elevation in the high-sensitivity (hs) troponin I level of 72.4 ng/L with a maximum level of 96.3 ng/L (normal range <16ng/L). Transthoracic echocardiography performed while at the ED had a poor acoustic window due to the patient´s biotype. It showed a hypertrophied left ventricle with normal-sized remaining cardiac chambers and an ejection fraction of 50% with hypokinesis of the inferior and basal parts of the septum. There were no valvular abnormalities, as well as no signs of pulmonary hypertension or right ventricular dysfunction. The patient was hospitalized with a non-ST elevation myocardial infarction (NSTEMI).

The patient was a nulliparous woman with a medical history of obesity (a BMI of 36 kg/m2), arterial hypertension diagnosed at age 25, and Cushing's disease due to an adrenocorticotropic hormone (ACTH)-producing pituitary tumor treated surgically 12 years before presentation. She did not have medical follow-ups or take corticosteroid supplementation for several years, without any reported symptoms. The patient was a non-smoker without any other cardiovascular risk factors or known history of established cardiovascular disease.

**Figure 2:**
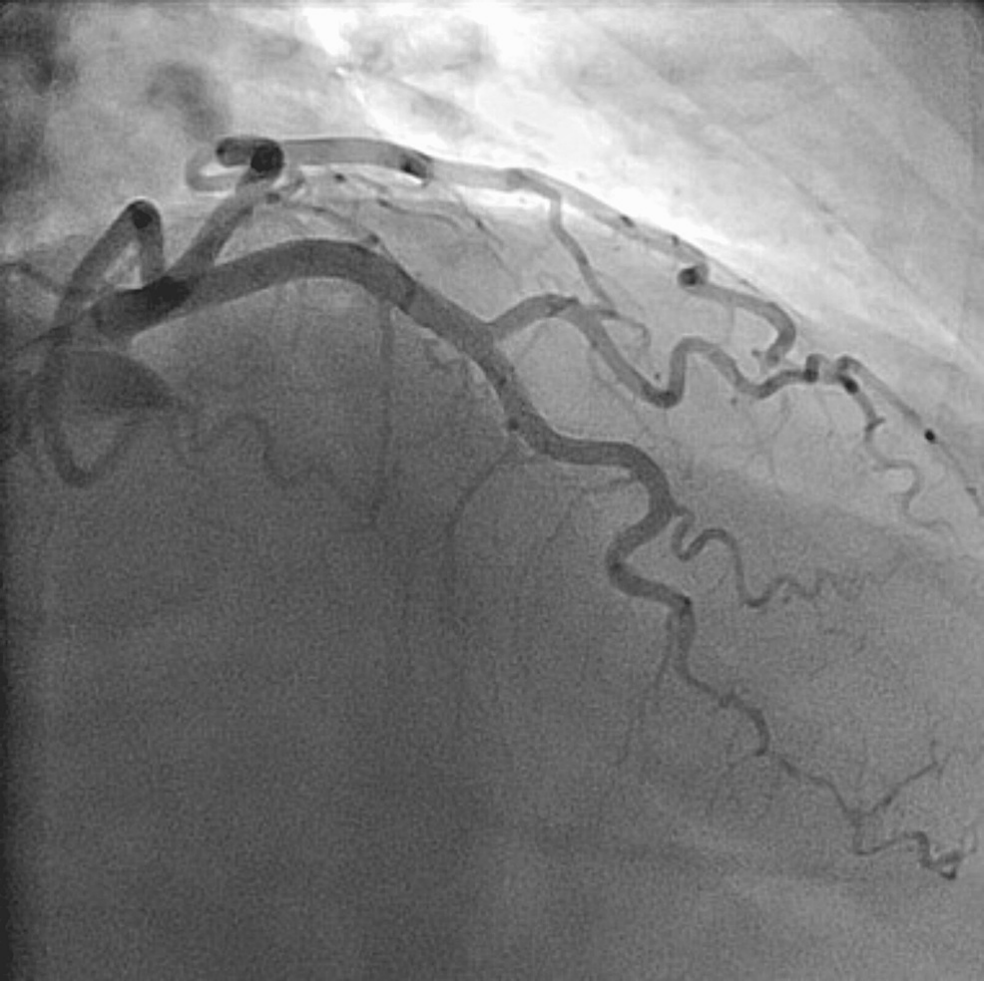
Cardiac catheterization showing type two spontaneous coronary dissection of the anterior descending coronary artery

She had a recurrence of chest pain in the emergency department without improvement after sublingual isosorbide dinitrate. Coronary angiography performed showed diffuse stenosis of the distal left anterior descending artery, compatible with a SCAD type two with thrombolysis in myocardial infarction (TIMI) II flow, as shown in Figure 2 and Video 1.

**Video 1:** Cardiac catheterization showing type two spontaneous coronary dissection of the anterior descending coronary artery

The patient was treated conservatively, with anticoagulation therapy during hospitalization and single antiplatelet therapy thereafter, without reported complications. Further investigations were performed to explore possible etiologies or associations with SCAD. Serum inflammatory, auto-immune, and immunologic workups were negative. Laboratory test results are shown in Table 1. An angiography-computed tomography scan of the abdomen and supra-aortic trunks showed no inflammatory changes or signs of fibromuscular dysplasia (FMD). There was no family history of SCAD, any evidence of hormonal treatment, or over-the-counter medications.

Chest pain recurred twice during hospitalization, with no electrocardiographic changes or elevation on hs-troponin I. Antianginal and antihypertensive therapy were titrated, and the patient was discharged on day seven.

At the follow-up visit one month later, the patient reported a recurrence of class II angina, according to the Canadian Cardiovascular Society (CCS). Transthoracic echocardiography evaluation showed preserved left ventricular systolic function with persisting inferobasal septum hypokinesia. A tetrofosmin single photon emission computed tomography (SPECT) of the heart did not reveal any significant changes in myocardial perfusion. A pituitary MRI showed a residual pituitary microadenoma. There were no signs of hypercortisolism, as the midnight salivary cortisol and 24-hour urine cortisol were normal, as was the dexamethasone suppression test. Antianginal medications were titrated with the patient reporting clinical benefit.

## Discussion

Cushing's syndrome is not traditionally considered a risk factor for SCAD. However, it is characterized by the overproduction of cortisol and a higher rate of cardiovascular events [9,12,13]. It is classically related to hypertension, hyperglycemia, hypercholesterolemia, weight gain, and a prothrombotic state [9,14]. In the literature, there are case reports of aortic and vertebral arterial dissections associated with hypercortisolism [7,8,10] or even SCAD in patients treated with high-dose corticosteroids [15]. Although the aorta differs structurally from coronary arteries, many of the components of the arterial wall, such as elastic and collagen fibers, are similar [13], which may predict similar effects of cortisol throughout the vascular territory.

Considering this association and after the exclusion of SCAD's common causes and associations, we hypothesized that the previous medical history of Cushing's disease has contributed to SCAD. The pathophysiologic relationship between these two conditions is plausible if we consider the vascular effects of cortisol referred to above.

This association may be of clinical relevance, and patients presenting with SCAD, particularly women, may be tested for overt or even subclinical forms of hypercortisolism. To the best of our knowledge, there are no other reported cases in the literature of Cushing's syndrome associated with SCAD.

## Conclusions

SCAD is an important cause of acute myocardial infarction and may be the first manifestation of an underlying systemic arteriopathy that leaves the patient vulnerable to dissection when exposed to a stress factor. The underlying mechanism of SCAD is not fully understood, and in addition to well-established causes such as connective tissue disease, postpartum, fibromuscular dysplasia, and hormonal therapy, Cushing's syndrome or other forms of hypercortisolism may contribute to this phenomenon.
